# Deciphering the killer‐cell immunoglobulin‐like receptor system at super‐resolution for natural killer and T‐cell biology

**DOI:** 10.1111/imm.12684

**Published:** 2016-12-14

**Authors:** Vivien Béziat, Hugo G. Hilton, Paul J. Norman, James A. Traherne

**Affiliations:** ^1^ Laboratory of Human Genetics of Infectious DiseasesNecker BranchINSERM U1163ParisFrance; ^2^ Imagine InstituteParis Descartes UniversityParisFrance; ^3^ Departments of Structural Biology and Microbiology & ImmunologyStanford UniversityStanfordCAUSA; ^4^ Department of PathologyUniversity of CambridgeCambridgeUK

**Keywords:** expression, haplotypes, killer‐cell immunoglobulin‐like receptors, ligands, natural killer cell, polymorphism

## Abstract

Killer‐cell immunoglobulin‐like receptors (KIRs) are components of two fundamental biological systems essential for human health and survival. First, they contribute to host immune responses, both innate and adaptive, through their expression by natural killer cells and T cells. Second, KIR play a key role in regulating placentation, and hence reproductive success. Analogous to the diversity of their human leucocyte antigen class I ligands, KIR are extremely polymorphic. In this review, we describe recent developments, fuelled by methodological advances, that are helping to decipher the KIR system in terms of haplotypes, polymorphisms, expression patterns and their ligand interactions. These developments are delivering deeper insight into the relevance of KIR in immune system function, evolution and disease.

**Abbreviations**
HCMV: human cytomegalovirus
HIV: human immunodeficiency virus
HSV: herpes simplex virus
KIR: killer‐cell immunoglobulin‐like receptors
NK cell: natural killer cell

## Introduction

**Figure 1:**
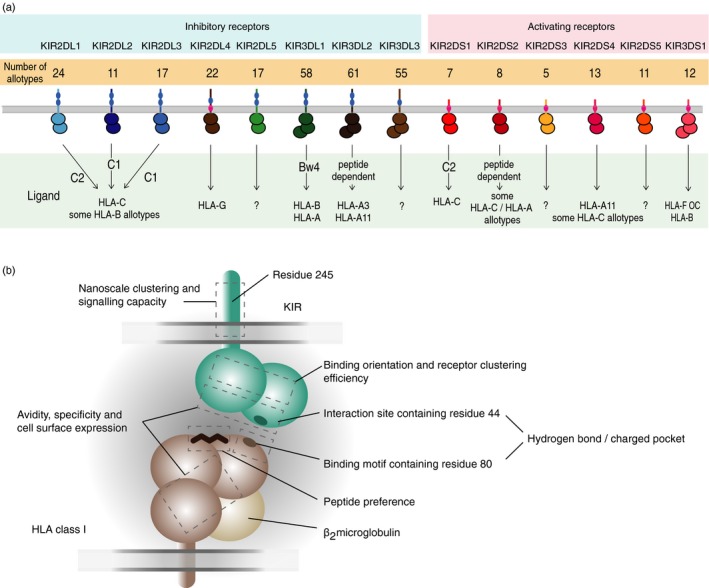
Killer‐cell immunoglobulin‐like receptors (KIR) proteins and their ligand interactions. (a) KIR have either two or three immunoglobulin‐like extracellular domains, KIR2D or KIR3D, respectively. They are either activating or inhibitory depending on the structure of their intracellular domain. Inhibitory KIR have long cytoplasmic tails (KIR**L*) that contain immunoreceptor tyrosine‐based inhibitory motifs (ITIM) that transduce inhibitory signals to the natural killer (NK) cell. Activating KIR have short cytoplasmic tails (KIR**S*) with a positively charged amino acid residue in their transmembrane region. The charged residue allows KIR proteins to associate with the TYROBP (DAP12) transmembrane signalling polypeptide, which acts as an activating signal transduction element because it contains an immunoreceptor tyrosine‐based activation motif (ITAM) in its cytoplasmic domain. KIR3DL1 and KIR3DS1, which are encoded by alleles of the same gene, *KIR3DL1/S1*, thus have opposing functions. KIR differentially bind HLA‐A, ‐B or ‐C allotypes and dimorphisms in the HLA class I *α* domains are the major determinants for this interaction. The binding motifs are referred to as C1 and C2 in HLA‐C and Bw4 in HLA‐B and HLA‐A. The precise KIR binding motif of HLA‐A*11, which can be recognized by KIR2DS2, KIR2DS4 and KIR3DL2, has not been determined.^10^, ^11^ Interactions may also be sensitive to polymorphism outside the HLA and KIR binding motifs and to the presented peptide sequence. The ligands for activating KIR and some inhibitory KIR are presently not well‐defined. OC, open conformers (b) Schematic to show how polymorphism in different parts of the KIR and HLA class I molecules diversifies their interactions. Key residues are KIR position 44 and HLA position 80, which control specificity and KIR position 245 that influences inhibitory signal strength, as discussed in the text.

Killer‐cell immunoglobulin‐like receptors (KIRs) are type I transmembrane glycoproteins belonging to the immunoglobulin superfamily. They are primarily expressed on natural killer (NK) cells but they are also expressed on subsets of CD4, CD8 and *γδ* T cells.^1^, ^2^, ^3^, ^4^, ^5^, ^6^, ^7^, ^8^ Comprising both activating and inhibitory forms they represent an archetypal paired receptor system.^9^ The best characterized ligands for KIR are HLA class I molecules that express either the Bw4, C1 or C2 motif (Fig. 1).

The functional activity and development of KIR‐expressing lymphocytes are modulated by interactions between these receptors and their ligands.^12^, ^13^, ^14^ A major function of circulating cytotoxic NK cells is to recognize and eliminate cells that fail to express self HLA class I molecules in the surveillance for virus‐infected or transformed cells.^15^, ^16^ By contrast, a major function of non‐cytotoxic NK cells in the uterus is to secrete cytokines to regulate placentation during pregnancy. This occurs through a mechanism of maternal allogeneic recognition involving interaction between KIR on maternally derived uterine NK cells with HLA on fetally derived cells.^17^ The KIR system acts to diversify NK cell stimulation potential through specificity of interaction and strength of signalling. In this regard, weakly inhibitory KIR/HLA combinations permit a lower threshold for cell activation than do strongly inhibitory KIR/HLA combinations.

**Figure 2:**
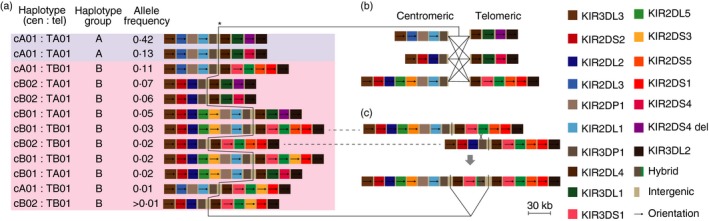
Structural haplotypes of the *KIR* gene cluster and recombination mechanisms. Numerous killer‐cell immunoglobulin‐like receptor (KIR) haplotypes with different gene content have been described. These haplotypes have been generated through serial duplications and deletions of chromosomal segments containing KIR genes. The distinction between alleles and genes is, therefore, sometimes blurred; for example *KIR2DS3* can be located in two different positions within the KIR locus. (a) The arrangements of genes in 12 common European haplotypes^18^ are shown. Typically, a person inherits between 14 and 24 *KIR* genes (between 7 and 12 KIR genes per haplotype). *KIR2DP1* and *KIR3DP1* are pseudogenes. Two broad haplotypes exist – *A* (light blue background) and *B* (pink background), resulting in genotypes that are an ‘AA’, ‘AB’ or ‘BB’. *A* haplotypes have a single arrangement of seven expressed genes that encode mostly inhibitory KIR, which are diversified by allelic variation. *B* haplotypes have varied gene arrangements and tend to comprise more activating genes and less allelic diversity. The *A* haplotype can be divided into two types depending on whether the *KIR2DS4* gene is full‐length (*KIR2DS4*) or carries a frameshift deletion (*KIR2DS4 del*). (b) Diversity has been generated by homologous recombination, particularly at a recombination hotspot (*) centrally sited within the gene cluster,^19^ which has shuffled the centromeric (cen) and telomeric (tel) parts of the locus encompassing allelic and gene‐content motifs. (c) Further diversity has been generated through continuing cycles of unequal crossing‐over (non‐allelic homologous recombination), which result in re‐assortment and addition or subtraction of genes in a ‘cut & paste’‐like manner.^20^, ^21^ So called fusion genes composed of parts of different *KIR* genes have been generated by unequal crossover events when the recombination has occurred within genes.^21^, ^22^

*KIR* genes are located in the leucocyte receptor complex on human chromosome 19q13.4. The genes are variably present in the germline between individuals, forming haplotypes with diverse gene content (Fig. 2), and numerous alleles exist for many of the genes. Despite the major implications of KIR variation for human health it is known that genome‐wide studies have poorly captured the diversity at the *KIR* locus. Through focused analyses, constituent polymorphism has been described at the basic levels – gene content of haplotypes, copy number, alleles and their frequencies. Resulting information has supported genetic, functional and disease investigation. In this review we discuss the outstanding challenges in KIR analysis and the recent methodological developments that are facilitating new discoveries.

## KIR genetics

### Functional consequences of *KIR* polymorphism

**Figure 3:**
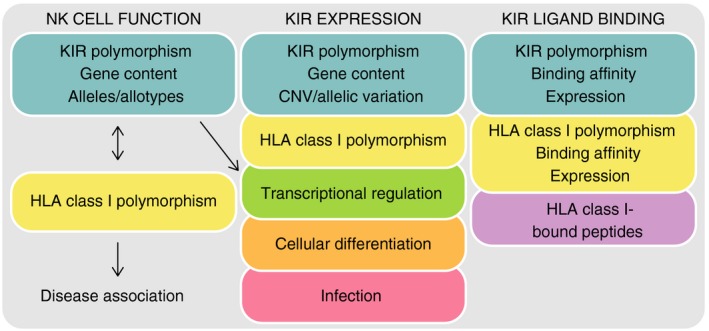
Major factors that influence natural killer (NK) cell function, expression and killer‐cell immunoglobulin‐like receptors (KIR) ligand binding.

Four influential discoveries cultivated the fundamental principle that genetic variation of *KIR* has a direct impact on NK cell function, and stimulated ongoing research into the impact of this variation on human health; (i) the *KIR* genomic region has variable gene content,^23^ (ii) *KIR* allelic variation affects KIR allotype function,^24^, ^25^ (iii) there are multiple alleles for each *KIR* gene^26^ and (iv) this genetic variation correlates with ability to control disease or reproduce^27^, ^28^, ^29^, ^30^ (Fig. 3). Even a single nucleotide mutation can dramatically change receptor expression,^25^, ^31^, ^32^, ^33^ ligand specificity^34^, ^35^, ^36^ or signalling strength. *KIR* gene copy number variation influences NK cell education, shaping the NK cell repertoire.^37^ Lastly, combinatorial diversity of *KIR* and *HLA* class I alleles also impacts NK cell function, because any given KIR allotype has differential reactivity to the allotypes of its cognate HLA class I ligand.^38^, ^39^, ^40^, ^41^, ^42^, ^43^ In this scenario one may expect specific alleles or combinations to be beneficial for resisting specific infections, or stimulating immune‐mediated disease.^44^ This combinatorial diversity is amplified by hosting the *KIR* and *HLA* genes on separate chromosomes, and the result is many millions of different cognate *KIR*/*HLA* class I genotypes, tending to individuality.^45^, ^46^ Hence, through evolving multiple genetic mutations in the *KIR* locus, the human population probably generates and maintains considerable diversity in immunity to evolutionarily nimble and diverse pathogens.^17^ That there is little in common between human, chimpanzee and orang‐utan *KIR* loci,^47^, ^48^ and that distributions of *KIR* genes track with ethnicity and geography^49^, ^50^, ^51^ are testament to this hypothesis of rapid evolution.

### KIR diversity and balancing selection

The interest in *KIR* genetic variation was piqued from early studies involving relatively small numbers and simple methods. The reason is that multiple diverse genotypes were detected in few individuals^23^, ^26^, ^52^ and the only plausible explanation was a highly heterogeneous genetic system with multiple common variants. These early studies therefore provided the first evidence that KIR may be subject to natural selection that maintains high diversity, just like it does for HLA.^19^, ^53^ The Yucpa population from Venezuela has low genomic diversity as a consequence of serial founder effects.^54^ There are only two common *KIR* haplotypes in the Yucpa (one *A* and one *B*; see Fig. 2 for nomenclature), but between them they carry all of the expressed *KIR* genes.^55^ This situation is extremely unlikely without the impact of a form of natural selection called balancing selection, which maintains genetic variation of specific loci in the population.^55^ Indeed, all human populations studied to date have a representation of *KIR A* and *B* haplotypes^51^, ^56^ and the prevailing hypothesis is that the *A* haplotypes are good for fighting infection^57^, ^58^ whereas *B* haplotypes are more beneficial for reproduction.^17^, ^59^

**Figure 4:**
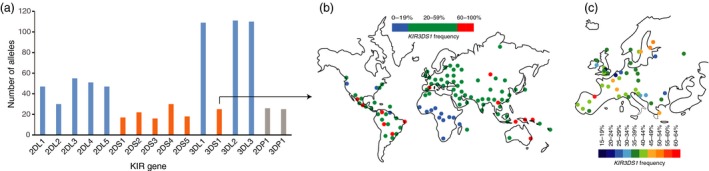
*KIR* allele counts and *KIR3DS1* frequency worldwide plots. (a) Number of alleles reported in the December 2015 release of the IPD KIR database.^60^ The advent of killer‐cell immunoglobulin‐like receptor (KIR) analysis by next‐generation sequencing is rapidly increasing the number of recognized KIR alleles.^68^ Blue are genes encoding inhibitory KIR, orange activating *KIR* and grey are pseudogenes. There are 753 alleles in total. (b) As an example of how *KIR* gene frequencies can vary significantly across populations, *KIR3DS1* gene frequency across the world and (c) Europe are shown (data from The Allele Frequency Net Database^56^).

The impact of balancing selection is also seen clearly in the DNA sequences of *KIR* genes.^19^, ^38^ Namely, a greater number of common sequences, and greater divergence between them is present in the *KIR* locus than would be expected if there were no selection, and when compared with other parts of the genome. A good example is *KIR3DL1/S1*, which has over 100 alleles characterized (Fig. 4)^60^ and has three divergent allele lineages that have been maintained by balancing selection for millions of years.^61^ A synergy of population genetics, phylogenetic analysis and comparison of nucleotide substitution rates among codons showed that this diversity is focused towards the parts of the KIR molecule that bind the HLA class I and peptide.^61^ The prediction that these major lineages of KIR3DL1/S1 have distinct ligand HLA/peptide‐binding properties has been borne out with crystallographic and functional studies.^35^, ^39^, ^41^, ^42^, ^62^, ^63^, ^64^, ^65^ Expansion of the phylogenetic analyses to include other KIR molecules revealed natural selection has consistently been focused towards residues that affect interaction with the HLA class I ligand,^66^ as well as those that affect the signalling properties of the receptor.^67^

### KIR and HLA co‐evolution

**Figure 5:**
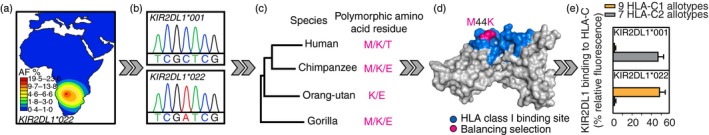
From discovery to function. Amino acid residues that have been subject to balancing selection can have dramatic effects on HLA class I recognition. Multiple novel *KIR* alleles may be discovered during population studies, and molecular analysis identifies the most functionally important. An example is KIR2DL1*022, which was discovered in the southern African KhoeSan population (a) and which differs from its parental allele KIR2DL1*001 by a single nucleotide substitution in codon 44 (b).^34^ Phylogenetic analyses that included the most closely related *KIR* from other hominoid species identified that residue 44 has been subject to balancing selection (c).^66^ Residue 44 occurs at the HLA binding site in the D1 protein domain of the killer‐cell immunoglobulin‐like receptors (KIR) molecule (d) (*PDB: 1IM9*).^70^ Substitution of methionine 44 (KIR2DL1*001) for lysine 44 (KIR2DL1*022) switches the specificity of the receptor from HLA‐C2 to HLA‐C1 (e). The methodological pipeline described above links population‐based analyses to functional mapping through sequence/phylogeny analysis and structural biology.

Specific combinations of cognate KIR and HLA class I ligand correlate in frequency across the world, indicating that co‐evolution between them continues in modern humans.^69^ Again, high‐resolution analysis of the Yucpa population yields further insight into this phenomenon. The population has extremely high frequency of HLA‐C7 and corresponding high frequency of a KIR2DL3 allotype, unique to the Yucpa, having reduced C7 binding.^55^ By comparison, the KhoeSan population from Southern Africa has one of the highest aggregate frequencies of HLA‐C allotypes expressing the C2 motif. Here, a KIR allotype that switched binding specificity entirely from C2 to C1 has evolved.^34^ Both of these binding changes are due to substitutions at residues subject to balancing selection (Fig. 5).^66^ Hence, in both populations it appears that KIR have been able to respond rapidly and specifically to HLA class I frequency changes (of unknown aetiology). In contrast, in the Māori of New Zealand, a very low frequency of HLA‐B allotypes expressing the Bw4 motif (again of unknown aetiology) has been countered by an increase in frequency of HLA‐A allotypes that express KIR ligands.^46^ Such studies of human populations at high resolution reveal functionally important changes occurring on a fine scale, the sum of which throughout human evolution has resulted in the strong signature of balancing selection throughout the *KIR* locus.

### KIR allelic variation

There is little doubt that the most basic level of presence/absence of *KIR* impacts NK cell activity, and that the number of those genes present can influence NK cell development^37^ or control of disease.^71^ However, the prevailing theme of the genetic studies to date is that analysis of gene content identifies the fundamental tenets, which are then refined following higher‐resolution analyses of the alleles. This stems from the realization that *KIR* alleles have different magnitudes of effect, creating hierarchical series of phenotypes. From this, testable hypotheses can be formed about the extent to which each gene or allele, in conjunction with ligand variation, can predispose to a phenotype. For example, the cell surface expression of KIR3DL1 and the ability to recognize HLA‐Bw4 ligand vary according to allele, and hence *KIR3DL1* alleles should be analysed accordingly in the disease context.^25^, ^38^, ^58^, ^72^ Similarly, polymorphism in the extracellular domain of KIR2DL1 influences the binding affinity to HLA‐C2.^34^, ^40^ Over 200 associations of *KIR* genes with disease resistance or susceptibility are published,^56^, ^73^ and the handful studied at high resolution are proving informative.^58^, ^74^, ^75^, ^76^ The field is therefore ripe for harvesting new information through in‐depth analyses.^68^, ^77^

### KIR imputation

A statistical model that infers *KIR* genotypes from whole‐genome single nucleotide polymorphism data has been developed with about 97% accuracy for the majority of *KIR* genes, and can distinguish the broad A/B haplotypes.^77^ This method overcomes many of the obstacles caused by the complexity of gene content diversity to enable efficient *KIR* disease association analyses in large cohorts, and access to the wealth of previously generated single nucleotide polymorphism array data.^78^

### KIR disease association studies

**Figure 6:**
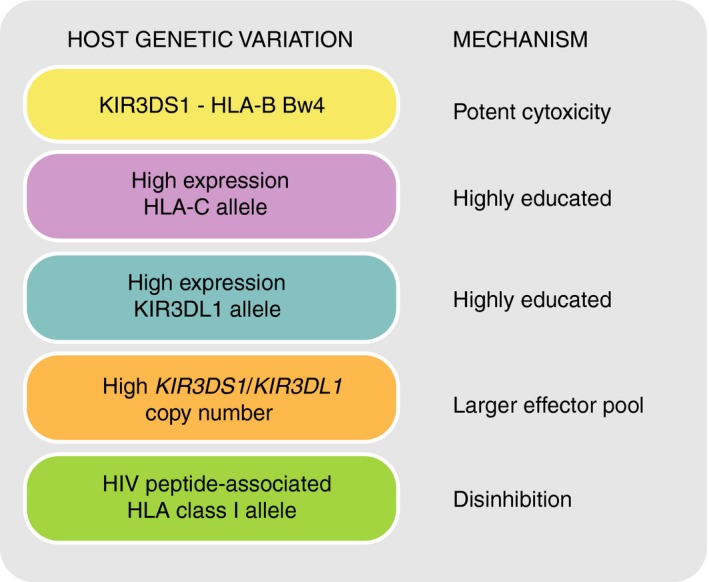
Host genetic variation and functions of HLA class I and killer‐cell immunoglobulin‐like receptors (KIR) in human immunodeficiency virus type 1 (HIV‐1) resistance. HIV‐1 down‐regulates HLA class I expression on the surface of infected CD4^+^ T cells so as to evade CD8^+^ T‐cell lysis.^86^, ^87^ However, this action exposes the infected cells to recognition and lysis by natural killer (NK) cells through KIRs. The effectiveness of an NK cell response, and consequently the outcome of infection, are linked to the host's KIR genes, their copy number and the expression levels of their ligands. More specifically, resistance to HIV‐1 correlates with; (i) the compound genotype, *KIR3DS1* ‐ *HLA‐B Bw4* (with isoleucine at position 80); KIR3DS1 binds HLA‐F open conformers, which can be expressed on HIV‐infected activated CD4^+^ T cells.^88^, ^89^ In this case NK cells expressing KIR3DS1 could also degranulate more potently in response to HIV‐infected Bw4^+^ CD4^+^ T cells and suppress viral replication.^90^ (ii) HLA‐C alleles that confer high cell surface expression^91^, ^92^; this may occur because the higher‐expressing HLA alleles result in highly educated NK cells, in addition to more efficient presentation of HIV epitopes to cytotoxic T cells, (iii) high expression KIR3DL1 alleles in Bw4^+^ individuals; again this could be due to highly educated KIR3DL1^+^ NK cells with greater activation potential when the ligand is down‐regulated by HIV,^58^ (iv) Increased copy number of *KIR3DS1* alone or *KIR3DL1* in the presence of *HLA‐B Bw4*; probably related to the clonal distribution of KIR, whereby the frequency of NK cells expressing a given KIR correlates linearly with gene copy number, (v) HLA allotypes that form complexes with HIV peptides that bind weakly to inhibitory KIR or strongly to activating KIR. Through any of these mechanisms, NK cells can exert selection pressure on HIV through KIR.^63^, ^93^ Conversely, HIV peptides that complex with HLA allotypes and bind inhibitory KIR with high affinity represent NK cell ‘escape’ variants via inhibition of NK cell function.^94^, ^95^

**Figure 7:**
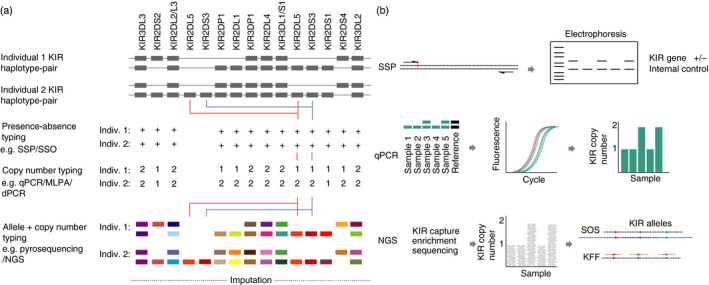
*KIR* genotyping methodology. (a) Representative haplotype‐pairs of two individuals are shown along with the expected results from different typing approaches; (i) presence/absence typing by PCR‐sequence‐specific primers (SSP) or sequence‐specific oligonucleotide (SSO) probes, (ii) copy number typing by quantitative PCR (qPCR),^96^ multiplex ligation‐dependent probe amplification (MLPA)^97^ and digital PCR (dPCR),^98^ (iii) allele and copy number typing by pyrosequencing and next‐generation sequencing (NGS), (iv) imputation infers *KIR* genotypes from single nucleotide polymorphism (SNP) data. (b) Schemes illustrating different typing approaches. SSP‐specificity relies on single nucleotide differences at the 3′ end of primers to distinguish different *KIR* genes. Real‐time qPCR combines SSP with fluorescently labelled probes to distinguish *KIR* genes and a reference gene (always two copies) amplifications in a multiplex reaction. *KIR* gene copy number is calculated by relative quantification. With NGS typing, oligonucleotide probes are used to capture the *KIR* genomic region. A bioinformatics pipeline converts sequence data into genotypes. Gene copy number is determined by relative read depth‐ratio of *KIR* genes compared with a reference gene (always two copies). Allele typing is achieved by filtering reads specific for genes based on alignment to all known reference alleles from *KIR* coding sequences (e.g. Son of Samtools (SOS)^68^). In parallel, sequence data can be probed with specific sequence search strings (‘*in silico* SSO’) to determine which alleles are present (e.g. KIR Filter Fish (KFF)^68^).

Combinations of *KIR* and *HLA* class I variants influence resistance to infections, susceptibility to autoimmune diseases and pregnancy syndromes, as well as outcome after haematopoietic stem cell transplantation (see The KIR and Diseases Database; Table 1).^79^ The influence of HLA class I and *KIR* gene variation on human immunodeficiency virus (HIV) disease outcome has been particularly well studied (Fig. 6).^80^ The variation at these genes has been described as a double‐edged sword because a particular genotype that confers protection from one disease (e.g. infection) could bestow increased risk to another disease type (e.g. autoimmunity or cancer).^67^, ^69^, ^81^ Population stratification is an important consideration in *KIR* association studies. It becomes problematic to analyse genes under strong selection and rapid evolution, such as the *KIR*, in admixed (mixed ancestry) populations where gene frequencies can vary significantly between subpopulations (Fig. 4). Approaches for *KIR* genetic analysis are improving in resolution and throughput (Fig. 7). Statistical developments offer additional strategies, such as principle component analysis of ancestry‐informative markers in case–control analysis to address admixture issues.^82^

**Table 1:** *KIR* and *HLA* databases and resources

Immuno Polymorphism Database	www.ebi.ac.uk/ipd/
Allele Frequency Net Database	www.allelefrequencies.net/
dbMHC and dbLRC	www.ncbi.nlm.nih.gov/gv/mhc/
	www.ncbi.nlm.nih.gov/gv/lrc/
LRC Haplotype Project	vega.sanger.ac.uk/info/data/LRC_Homo_sapiens.html

The Immuno Polymorphism Database (IPD) provides a repository for killer‐cell immunoglobulin‐like receptors (KIR) sequences and includes, allele alignments, fully sequenced KIR haplotypes, donor KIR B‐content group calculator, allele ethnicity tool and primer/probe search tool.^83^ The Allele Frequency Net Database (AFND) provides a repository for KIR allele frequencies and listings of KIR disease associations.^56^ dbMHC and dbLRC databases provides sequences and frequency distributions for alleles of the MHC and leucocyte receptor complex (LRC) as well as an alignment viewer, primer/probe search tool and typing kit interface.^84^ LRC Haplotype Project provides DNA sequence data from several human LRC haplotypes including contig maps, annotation and a catalogue of all single nucleotide polymorphisms and insertions/deletions.^85^

## KIR ligand binding

### KIR and HLA allotype interactions

Three key advancements have progressed understanding of the recognition of HLA class I by KIR; (i) the crystal structures of KIR in complex with their HLA class I ligands,^10^, ^35^, ^70^, ^99^ (ii) the development of soluble KIR^36^, ^100^, ^101^ and (iii) their use in a multiplex immunoassay against a broad panel of HLA class I allotypes^102^, ^103^ (Fig. 3). These tools have enabled fine‐scale analyses of KIR and HLA class I interaction diversity.

Two‐immunoglobulin domain inhibitory KIRs (KIR2DL1‐3) bind HLA‐C (Fig. 1). Originally considered separate genes, *KIR2DL2* and *KIR2DL3* are now known to segregate as alleles of the same gene, termed *KIR2DL2/3*. Initial studies indicated a simple bipartite system in which KIR2DL2/3 recognizes HLA‐C allotypes with asparagine at residue 80 (the HLA‐C1 motif), and KIR2DL1 recognizes HLA‐C allotypes with lysine at residue 80 (the HLA‐C2 motif). Dimorphism at residue 44 of the KIR molecule causes these specificity differences, where KIR2DL2/3 has lysine and KIR2DL1 has methionine.^36^, ^100^, ^104^, ^105^ Crystal structures showed that K44 of KIR2DL2 forms a hydrogen bond with the N80 of HLA‐C1.^99^ By contrast, M44 of KIR2DL1 has no direct contact with HLA‐C but forms part of a charge pocket that accommodates the K80 of HLA‐C2.^70^ Other residues are involved in the interaction however, and their polymorphism means that there is a range of binding characteristics determined by KIR allotype. The interactions of KIR2DL are further diversified by polymorphism within the subsets of C1‐bearing and C2‐bearing HLA allotypes.^34^, ^103^ The basis for these hierarchies may occur due to polymorphism at sites other than position 80, or the distinct repertoires of peptide presented by the different HLA‐C allotypes.^106^ An additional feature of HLA‐C polymorphism is the differential cell surface expression exhibited by individual allotypes,^91^ although how this variation impacts NK cell reactivity is yet to be fully defined.

KIR2DL2/3 allotypes form a continuum of binding strength and specificity that includes several inactivated or weakened variants.^34^, ^40^, ^55^, ^107^, ^108^, ^109^ Studies using soluble KIR showed that allotype KIR2DL3*001 is C1‐specific and binds relatively weakly, whereas KIR2DL2*001 binds more strongly and has cross‐reactivity with C2.^103^ In addition to those directly at the binding site, the substitutions that cause these functional differences may affect the angle or orientation of the binding domain,^55^, ^103^ or the efficiency of receptor clustering at the cell surface.^99^, ^108^ Allelic polymorphism also influences the functional properties of KIR2DL1, which has evolved to be a highly specific receptor for HLA‐C2.^110^ Yawata *et al*. first reported that NK cells educated via KIR2DL1*004 had lower interferon‐*γ* production than those educated via KIR2DL1*003.^111^ This occurs because KIR2DL1*004 has weak affinity for HLA‐C2 targets^34^ as well as reduced capacity for intracellular signal generation.^112^ A single dimorphism in the transmembrane region determines the signalling capacity; allotypes with R245 transduce a functional inhibitory signal and those with C245 have reduced inhibitory signalling.^112^ Super‐resolution microscopy has revealed that the nanoscale organization of KIR at cell surfaces depends on the transmembrane sequence, which in turn affects downstream signalling.^113^ Further modifying the functional range of KIR2DL1 are polymorphisms in the extracellular and cytoplasmic domains that regulate avidity and specificity, as well as the level of cell‐surface expression (Fig. 1).^34^, ^40^

Like the inhibitory KIR2D, the interactions between inhibitory KIR3D and their cognate ligands are diversified by their considerable polymorphism. KIR3DL1 recognizes *HLA‐A* and *HLA‐B* alleles that encode the Bw4 motif, a region that spans residues 77–83 on the *α*‐1 helix of the molecule (Fig. 1).^35^, ^114^, ^115^ Recognition of Bw4 by KIR3DL1 is sensitive to polymorphism both within and outside the Bw4 motif, as well as to the sequence of the bound peptide.^42^, ^63^, ^116^, ^117^, ^118^ Early work showed that HLA‐Bw4 allotypes with I80 formed more potent ligands for KIR3DL1 than those with T80,^119^ a functional difference reinforced by associations with disease outcome.^27^, ^58^, ^120^, ^121^, ^122^ However, recent high‐resolution studies have identified several I80 Bw4 allotypes that are poorly recognized by KIR3DL1, providing weaker KIR3DL1 ligands than selected T80 Bw4 allotypes.^39^, ^41^, ^123^ Compounding the difficulty of understanding the interactions between KIRD3DL1 and Bw4 is the extensive functional polymorphism of *KIR3DL1*, which changes its cell‐surface expression^25^, ^38^ and capacity to recognize the Bw4 epitope.^41^, ^65^, ^123^

### Activating KIR ligand interactions

Although disease associations^27^, ^76^, ^124^, ^125^ and sequence homology^67^ with their inhibitory counterparts suggest that activating KIR recognize HLA class I, their cognate ligands have been harder to identify (Fig. 1). As the body of research dedicated to activating KIR has grown, it has become clear that three conserved features of their biology are likely to have hampered the process of ligand discovery; (1) their low affinity for HLA class I,^11^, ^40^, ^105^, ^126^, ^127^ (2) narrow specificity^11^, ^126^, ^127^, ^128^ and (3) high peptide selectivity.^10^, ^11^, ^64^, ^127^ These features are epitomized by KIR2DS2*001, which binds weakly to C1‐bearing HLA‐C*16:01 and HLA‐C*03:02^126^, ^127^ and recognizes HLA‐A*11:01 in a peptide‐dependent manner.^10^ Another possibility is that activating KIR recognize virus‐induced ligands^129^, ^130^ or altered self‐HLA class I molecules caused by viral infection. Recent work has identified open conformers (not bound to *β*_2_‐microglobulin or peptide) of non‐classical HLA class I molecule, HLA‐F, as ligands of KIR3DS1.^88^, ^89^ Because HLA‐F can be expressed on activated or HIV‐1‐infected lymphocytes, KIR3DS1‐HLA‐F interaction could be important in the control of the T‐cell response^131^ and/or HIV‐1 infection.

Activating KIR are less polymorphic than inhibitory KIR (Fig. 4).^132^ However, the exploration of functional allotypic variation in activating KIR is in its infancy, and several studies point to its potential importance. Examples include the observation that KIR2DS1 allotypes recognize C2‐bearing HLA‐C with a range of avidities^40^ and that KIR3DS1*014, but not KIR3DS1*013, recognizes the HLA‐Bw4 epitope.^65^ Further, epidemiological studies suggest that specific variants of KIR2DS5, for which a ligand remains elusive, protect against the development of reproductive disorders.^76^, ^133^

Reporter systems could be useful tools to screen for activating KIR ligands and peptide influences of inhibitory KIR.^129^ The reporter cells are constructed to express the extracellular domains of activating KIR fused to the human CD3*ζ* cytoplasmic domain. Signalling through these hybrid receptors results in the expression of green fluorescent protein, which can be detected by flow cytometry. This method proved helpful to show that HLA‐F open conformers are ligands for KIR3DS1.^88^

### KIR peptide‐dependence

The emerging field of peptidomics has relevance to the KIR field because binding of KIR to their respective HLA class I ligand is peptide‐dependent.^134^ There are different mechanisms by which viral infection can rapidly and radically affect the HLA class I peptide repertoire. Some viruses have evolved to evade NK cell immunity through the selection of mutations in MHC‐presented peptides that enhance binding to inhibitory NK cell receptors including the C‐type lectin‐like CD94:NKG2A heterodimer receptor and KIR2DL3.^135^, ^136^ Conversely, virus‐induced changes in peptide repertoire may promote beneficial action through KIR by disrupting HLA class I recognition by inhibitory KIR, releasing constraint on NK cells to mount a positive clearance response to infected cells.^135^ Structural analysis of peptide interaction by NK receptors and better understanding of the mechanisms by which viruses evade the NK cell response could assist the development of novel targeted interventions to exploit the antiviral activities of NK cells.

## KIR gene expression

### NK and T‐cell KIR repertoire

With the exception of *KIR2DL4* and *KIR3DL3*,^137^, ^138^ *KIR* gene expression is clonally distributed and only a fraction of NK and T cells express a given KIR. KIR repertoire formation is complex. At least six factors are recognized to influence KIR repertoire; (i) transcriptional regulation, (ii) *KIR* gene content, (iii) allelic variation, (iv) cellular differentiation, (v) self‐HLA class I ligands and (vi) infection (Fig. 3).

KIR repertoire formation is governed by bi‐directional promoter activity and epigenetic silencing. High CpG methylation of the *KIR* proximal promoter was reported in NK cells and CD8^+^ T cells lacking expression of the corresponding KIR molecule, and vice versa.^139^, ^140^, ^141^ KIR expression requires the intermediate promoter Pro1;^142^ however, the strength of KIR proximal promoter antisense activity is probably responsible for clonal KIR distribution in NK and T cells.^7^, ^142^, ^143^, ^144^ The relative affinity of binding sites for transcription factors involved in sense versus antisense promoter activity determines the probability of generating the sense transcript required for gene activation.^142^, ^143^ A recent mouse study suggests that activating receptor‐mediated signalling might regulate this process during NK cell development.^145^ Polymorphisms in the *KIR* promotors also impact KIR expression.^142^, ^146^, ^147^

Beside epigenetic and transcriptional regulation, KIR repertoire formation is also dependent on *KIR* gene content and allelic variations. Each *KIR* gene is usually present in between zero and three copies in a given individual. As well as when the gene is absent, KIR expression is also abolished in individuals that are homozygous for a null allele, or who only carry the null allele of the gene – e.g. KIR3DL1*004 null allele is present at a mean frequency of 24·2% (SD 10·3) in worldwide populations.^56^ The frequency of cells positive for a given KIR is tightly linked to *KIR* gene copy number; a donor with two copies of *KIR3DL1* will have a greater frequency of KIR3DL1^+^ NK cells than donors with only one copy,^37^, ^38^, ^142^ suggesting that each *KIR* gene copy is regulated independently. Additionally, NK cell KIR expression is related to cellular differentiation. KIR expression is weak or absent in immature NKG2A^+^ CD56^bright^ NK cells and increases gradually with maturation, reaching its maximum level in NKG2A^−^ CD56^dim^ NK cells.^148^, ^149^, ^150^ Similar observation was made for T cells, in which KIR expression is virtually absent in CD4 and CD8 naive T cells and reaches its maximal level in differentiated effector memory T cells.^2^, ^5^, ^151^, ^152^ Accordingly, KIR expression is low in less mature cord blood NK and T cells compared with healthy adult control cells.^153^, ^154^

Unlike T‐ and B‐cell repertoire formation, there is no evidence for negative selection or deletion of NK cells expressing a combination of receptors that could be harmful or useless.^108^, ^155^ Instead, in a process termed education, only NK cells expressing self‐specific HLA class I inhibitory receptors (NKG2A or KIR) become fully functionally competent. The degree to which NK cell education shapes the KIR repertoire has been a matter of debate. Using a mathematical and phenotypic approach, Andersson *et al*. demonstrated that an adult's NK cell KIR repertoire formation was largely stochastic, in line with probabilistic expression of KIR under bidirectional proximal promoter activity and no selection.^156^ However, other studies showed a slight but significant bias of the global KIR repertoire in adults toward the expression of KIRs able to recognize self‐HLA class I.^111^, ^157^ Paradoxically, the same authors demonstrated that KIR repertoire in newborns was not biased toward self‐HLA class I recognition.^158^ This observation suggested that KIR repertoire acquisition was indeed stochastic but the slight bias observed in adults might be driven by infections encountered later in life.

In 2004, Guma *et al*. showed that expression of NKG2C, an activating receptor for HLA‐E, was increased in individuals infected with the human cytomegalovirus (HCMV) and that NKG2C^+^ cells expressed high level of KIRs.^159^ It was later demonstrated that these HCMV‐associated NKG2C^+^ NK cells express self‐specific KIRs and account for the vast majority of adult KIR repertoire deviation toward self HLA class I.^160^ HCMV‐adapted NK cells lacking NKG2C expression were also reported, most of them expressing activating KIRs.^160^, ^161^ Using a large NKG2C‐deficient cohort, it was recently demonstrated that adaptive NK cell responses could occur in the absence of both activating KIRs and NKG2C; however, these adaptive NK cells also largely display repertoire deviation toward self‐specific inhibitory KIR expression.^162^, ^163^ Also, self‐specific inhibitory KIR expression is not required for generating adaptive NK cells but is probably necessary for optimal functions. Indeed, HCMV^+^ TAP‐deficient individuals, with a considerable decrease of HLA class I expression at the cell surface, can develop NKG2C^+^ adaptive NK cells but these cells remain hypofunctional.^164^

**Figure 8:**
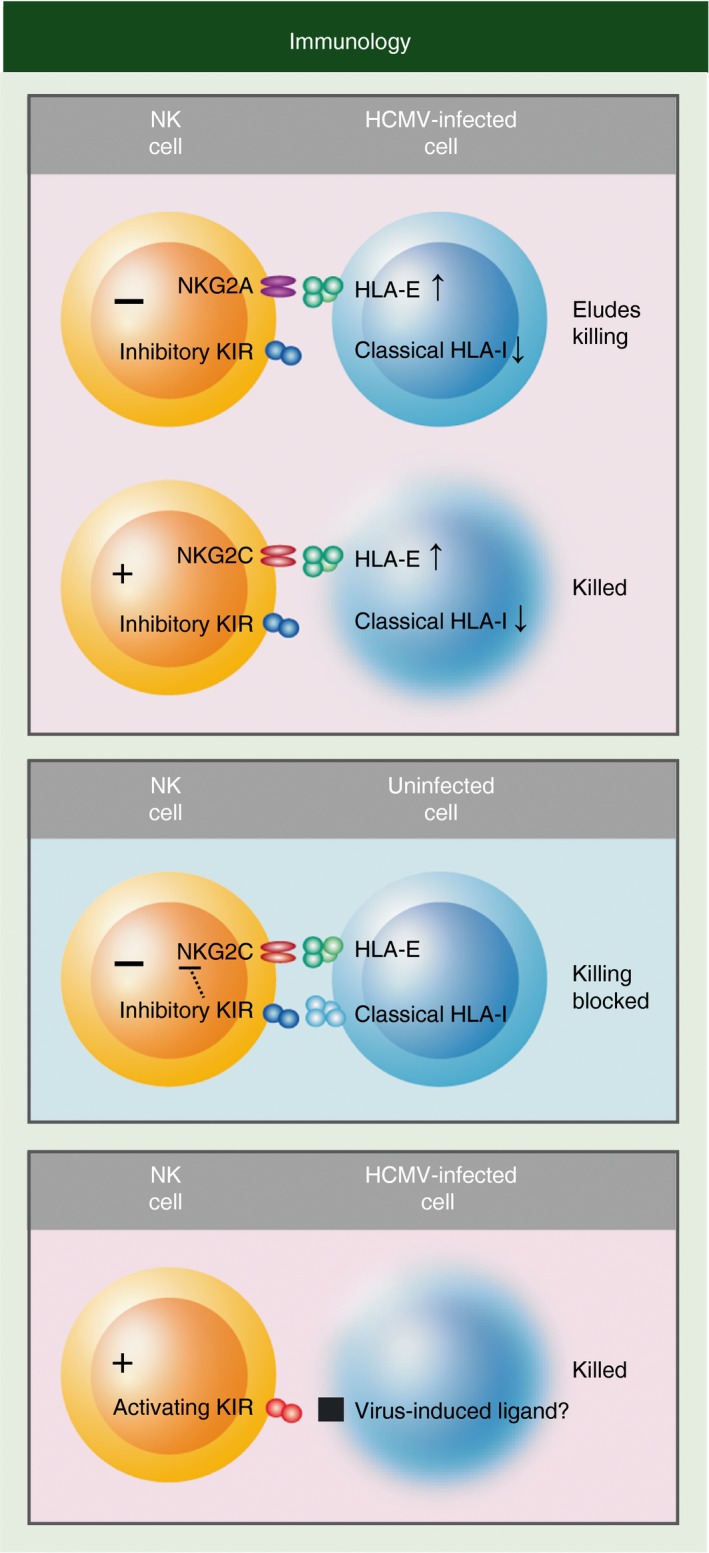
Natural killer (NK) cell strategies in human choriomeningitis virus (HCMV) infection. Viral proteins up‐regulate HLA‐E expression to selectively inhibit NK cells through the NKG2A inhibitory receptor. Concomitantly, the virus down‐regulates classical HLA class I expression to evade CD8^+^ T cells. NKG2C allows NK cells to detect HLA‐E^+^ HLA class I‐infected cells. Hence, ‘missing‐self’ (no HLA class I) triggers activation of NK cells already educated/licenced by self‐HLA class I molecules.^165^, ^178^ Non‐infected cells are protected from NK cell cytotoxicity by recognition of HLA class I by inhibitory killer‐cell immunoglobulin‐like receptors (KIR), which curbs activation through NKG2C. Activating KIR may directly recognize ‘altered‐self’ ligands that are induced by the virus.^15^

Studies reporting adaptive NK cell expansion in various viral infections including HIV, hepatitis B virus, hepatitis C virus, chikungunya virus and hantavirus show that adaptive NK cells only occur in HCMV^+^ individuals, perhaps as the result of opportunistic viral reactivation.^165^, ^166^, ^167^, ^168^ To date, apart from HCMV, no other viruses have been seen to correlate with the appearance of adaptive NK cells, and associated KIR repertoire deviations, including herpes simplex virus (both HSV‐1 and HSV‐2) and varicella‐zoster virus.^160^, ^169^ Instead, it was shown that acute Epstein–Barr virus infection induces NKG2A^+^ CD57^+^ expansion lacking KIR expression.^170^ Altogether, these findings suggest that KIR, and adaptive NK cells, might have mainly evolved to control HCMV. Supporting this hypothesis, HCMV developed several strategies to escape NK cell control.^171^, ^172^, ^173^ Viral proteins induce HLA‐E expression while decreasing classical HLA class I (HLA‐A, B and C) expression at the cell surface of infected cells, allowing the virus to selectively escape NKG2A^+^ NK cells and interfere with CD8^+^ T‐cell recognition, respectively.^174^, ^175^, ^176^, ^177^ NKG2C and inhibitory KIR come into play to counteract this escape strategy of HCMV. NKG2C allows adaptive NK cells to recognize HLA‐E^+^ HLA class I^−^ infected cells while self‐specific KIR prevent them killing HLA‐E^+^ HLA class I^+^ non‐infected cells (Fig. 8). This model provides an explanation for the skewed repertoire in adaptive NK cells seen in HCMV^+^ individuals towards inhibitory KIR that recognize self HLA class I. Activating KIRs could play a similar role to NKG2C and recognize unknown HCMV‐induced ligands, similarly to Ly49H/m157 in mouse.^129^, ^130^

The role of KIR expression in T cells remains unclear and most of the studies have focused on CD8 T cells, which contain the largest KIR^+^ subset within the T‐cell compartment. Although NK cell KIR repertoire is considered stable in time, the frequency of KIR^+^ T cells increases with age, due to accumulation of terminally differentiated T cells.^141^ Interestingly, HCMV‐specific CD8 T cells almost completely lack KIR expression, and the specificity of KIR‐expressing cells remains largely unknown.^2^, ^5^, ^7^, ^151^, ^179^ In contrast, it was shown that KIR^+^ CD4^+^ T cells display specificity against HCMV but not Epstein–Barr virus or HSV‐1.^4^ It is clear that inhibitory and activating KIR can, respectively, dampen and co‐stimulate T‐cell receptor‐mediated activation in CD4 and CD8 T cells.^5^, ^151^, ^179^, ^180^, ^181^, ^182^ Unlike NK cells, self‐specific inhibitory KIR expression does not educate T cells, as they do not display enhanced functional responses upon T‐cell receptor triggering.^7^ Instead, it was shown that *ex vivo* KIR^+^ effector memory CD8^+^ T cells were hyporesponsive to T‐cell receptor triggering compared with KIR^−^ effector memory CD8^+^ T cells.^7^ It was proposed that KIR expression protects from activation‐induced cell death in a ligand‐independent manner, perhaps explaining their accumulation with aging.^2^, ^183^

### KIR repertoire analysis

**Figure 9:**
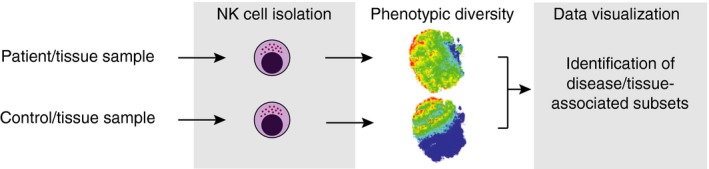
Natural killer (NK) cell analysis by mass cytometry by time‐of‐flight. By interrogation of multiple markers simultaneously, cell phenotypes can be compared between, for example, patients versus controls or between different tissue sites. The approach allows alterations in lymphocyte composition or abundance to be detected that are linked to killer‐cell immunoglobulin‐like receptors (KIR) genotypes and disease.

KIR repertoire analysis has proved important in the study of NK cell function. Future studies will need to systematically analyse in‐depth KIR repertoire of organ resident NK cells, which are known to display unique KIR repertoires, at least in the uterus and the liver.^184^, ^185^, ^186^ When studying the KIR repertoire all the parameters that are known to influence KIR expression should be considered (Fig. 3), most of all KIR genotypes and cellular differentiation markers. Cytometry panels require many colours to include as many KIR as possible, together with differentiation markers including at least NKG2A, NKG2C and preferably other markers specific to adaptive NK cells (e.g. FC*ε*R1*γ*, CD57 and NKp30). Strategies for KIR repertoire analysis using high‐dimensional FACS analysis were recently described.^187^ Several limitations remain in such analysis, such as mis‐binding of antibodies relating to amino acid substitutions in particular KIR allotypes. For example, anti‐KIR2DL3 ECM41 antibody does not recognize KIR2DL3*015 or KIR2DL3*005.^187^ These allotypes are recognized by antibodies with specificity for receptors encoded by other KIR genes (Table 2). New next‐generation sequencing techniques will allow fast and cost‐effective KIR typing at allelic resolution and will facilitate KIR repertoire interpretation.^68^ In addition, high‐dimensional mass cytometry (mass cytometry by time‐of‐flight) is a powerful tool for investigating NK cell repertoire diversity through the analysis of many cellular markers simultaneously^188^ (Fig. 9). The approach is being used to characterize the phenotypes of lymphocytes and their abundance in biopsies of patients, to gain insight into, for example, how receptor genotypes influence disease susceptibility in a cell‐type‐specific manner.^188^, ^189^, ^190^, ^191^ Once identified, discrete NK cell subpopulations associated with disease could be harnessed for immunotherapeutic strategies or may predict response to treatment. Combined with other single‐cell techniques, e.g. RNA‐Seq, this could be an important step forward to personalized and more cost‐effective treatment.

**Table 2:** Specificities and reported cross‐reaction of killer‐cell immunoglobulin‐like receptors (KIR) antibodies

Clone	Specificity	Reported cross‐reaction and (comments)	Reference
143211	KIR2DL1	KIR2DS5	192
HP‐3E4	KIR2DL1	KIR2DS1 and KIR2DS4	193
EB6	KIR2DL1, KIR2DS1	KIR2DL3*005	187, 194, 195
11PB6	KIR2DL1, KIR2DS1	KIR2DL3*005	194
HP‐MA4	KIR2DL1, KIR2DS1	KIR2DS3 and KIR2DS5	192, 193
GL183	KIR2DL2, KIR2DL3, KIR2DS2		196, 197, 198
DX27	KIR2DL2, KIR2DL3, KIR2DS2		52
CH‐L	KIR2DL2, KIR2DL3, KIR2DS2		199
180701	KIR2DL3	(Does not recognize KIR2DL3*005 and *015)	187
ECM41	KIR2DL3	(Does not recognize KIR2DL3*005 and *015)	194
UP‐R1	KIR2DL5		200
1F12	KIR2DS2, KIR2DL3		201
179315	KIR2DS4		140
JJC11·6	KIR2DS4	KIR2DS3	192
FES172	KIR2DS4		202
DX9	KIR3DL1		203
5·133	KIR3DL1, KIR3DL2	All KIRs except KIR2DS1 and KIR2DS3	192
Z27	KIR3DL1, KIR3DS1		204, 205, 206
DX30	KIR3DL1/KIR3DL2		207
DX31	KIR3DL2		207
NKVFS1	PanKIR2D		208

Sequence homogeneity between KIR genes at the genomic level translates to high similarity between encoded protein products. As such, analysis of KIR expression is not trivial in primary cells because of the cross‐reaction of available antibodies, in particular between activating and inhibitory isoforms. This can lead to misinterpretation of cell‐staining results. These cross‐reactions must be taken into account when analysing KIR expression. Staining experiments can be supported with control stains, references, representative stains on relevant donors and precise descriptions of strategies for differential staining using sets of antibodies.

Although the new methods are increasing scale and resolution of all aspects of KIR study, one obstacle that future repertoire studies will need to overcome is the current lack of antibodies that specifically recognize KIR2DS2, KIR3DS1, KIR2DS3 and KIR2DS5 (Table 2). Although antibody combinations can help to decipher these activating KIRs,^187^, ^192^, ^201^ they do not allow analysis at single KIR level because of antibody cross‐reactivity. Aptamers, short single‐stranded nucleic acid oligomers that bind to a specific target molecule, with their unique features of high binding affinity and specificity, could offer a useful alternative to antibodies in discriminating subtly different forms of KIR.^209^ Improving KIR repertoire analysis will contribute to our understanding of clinical situations where KIR have a proven or suspected role such as antiviral immune response, transplantation, autoimmunity and reproduction.

## KIR model systems

Mouse NK cells do not express KIR but instead Ly49 receptors (C‐type lectin‐like type II transmembrane disulphide‐bonded homodimers), which perform analogous functions.^210^ For example, like *KIR* genes, *Ly49* genes encode both activating and inhibitory NK receptors that regulate NK cell biology through binding MHC class I molecules. Despite differences between mice and humans with regard to the immune and reproductive systems, the mouse has been used to study how imbalance of NK cell inhibition or activation potential influences pregnancy.^211^ Humanized mice of homogeneous genetic background might in the future enable study of individual KIR and HLA class I variants in isolation.^212^ Using a mouse transgenic for an HLA‐B allele that encodes the Bw4 epitope, a recent study investigated the roles of cell‐intrinsic and cell‐extrinsic HLA class I molecules for educating human NK cells.^213^

Because mice and humans are so divergent, primates are used as *in vivo* models to study NK cell biology. Some non‐human simian species show a comparable level of diversity and complexity in *KIR* haplotypes to humans.^214^ However, there are extensive differences in size and organization between the *KIR* loci of higher primate species.^215^ It is evident in primates that different lineages of *KIR* genes have been expanded concomitantly with species‐specific evolution of *MHC* class I genes.^216^ In rhesus macaque, an important animal model of human diseases such as AIDS, binding of certain KIR is influenced by the same MHC epitopes (Bw4 and Bw6) that are important determinants of human KIR interactions.^217^ In the last decade, progress has been made in characterizing *KIR* genes in primates and developing specific genotyping assays.^218^, ^219^ Specific interactions between KIRs of primates and MHC class I ligands are being identified and monoclonal antibodies against primate KIR proteins are being generated.^220^, ^221^ These advances are enabling phenotypic characterization of KIR expression on NK cells and T‐cell subsets in primates and investigation of KIR‐MHC biology in primate models of infectious disease.^222^, ^223^, ^224^

## Therapeutic intervention

As KIRs are expressed on effector cells such as adaptive NK cells and effector memory T cells, which have undergone clonal expansion, it is not surprising that KIRs are often found expressed on malignant cells. For instance, in NK and T‐cell large granular lymphocytosis lymphocytes often express activating or inhibitory KIRs in a clonal manner.^225^, ^226^, ^227^, ^228^ Patients with Sezary syndrome typically display CD4^+^ T cells expressing KIR3DL2, which might contribute to disease onset.^228^, ^229^ For this reason, KIR are considered as a potential therapeutic target because they are only expressed on a small subset of normal lymphocytes, the deletion of which is unlikely to be harmful.^230^

Genetic information on KIR and HLA is already being used clinically to choose donors for haematopoietic stem cell transplantation for optimal outcome.^231^ Indeed, it is estimated that a significant reduction of relapse after transplantation for acute myeloid leukaemia can be achieved by choosing donors based on their KIR and HLA class I genotype.^232^, ^233^ This could be extended in the future to help, for example, sperm donors with the lowest risk of adverse pregnancy outcomes in assisted reproduction. In‐depth understanding of the molecular pathways that control NK cells will be critical to the therapeutic manipulation and in adoptive transfer strategies of these powerful lymphocytes,^234^ such as in cancer immunotherapy.^16^, ^29^, ^235^, ^236^ Immune‐modulation therapies that alter NK and T‐cell function are already in development. For example Lirilumab, the monoclonal antibody that binds KIR, is currently in a phase II clinical trial for lymphoma.^237^ By blocking the interaction of inhibitory KIR with their HLA class I ligands this antibody facilitates activation of NK cells by impeding inhibitory signalling, potentially promoting destruction of tumour cells.

## Conclusion

Analysis of complex genomics represents a significant new frontier for immunology. Challenges lie in determining allelic copies precisely and developing molecular and computational strategies to analyse them at the scale required to definitively relate them to phenotypes. For the *KIR* gene cluster this process is underway and yielding valuable insights. KIR, therefore, provide a useful model for developing analysis methods for other families of proteins shaped by multi‐allelic copy number variation, immune‐related or otherwise. An important advance has been the development of targeted techniques and bioinformatics tools to precisely type and analyse *HLA* and *KIR* copy number and alleles with great accuracy and in high sample numbers. Understanding how *KIR* variation influences the initiation and progression of disease will be achieved through the application of these novel methods in multidisciplinary projects involving geneticists, statisticians, structural biologists, immunologists and clinicians. The overarching question is how the signals from lymphocyte receptor interactions determined at the genetic level translate to differing functionality and outcomes in settings of infections, pregnancy, autoimmunity and cancer. Understanding the cellular and molecular mechanisms underlying the KIR‐HLA system will contribute to the development of interventions and therapies to ameliorate adverse consequences when these mechanisms go awry.

## Summary

The advent of next‐generation sequencing is set to allow the determination of *KIR* and *HLA* sequences at super‐resolution. Combining such studies with high‐resolution functional mapping of these polymorphic genes will provide unprecedented insight, not only into the molecular mechanisms that govern the interactions between receptor and ligand, but also into the pathophysiology of infectious and non‐infectious disorders in which KIR and HLA play critical roles.

## Disclosures

The authors declare that there is no conflict of interest regarding the publication of this paper.
